# Incidence Rates and Risk Ratios of Normal Tension Glaucoma in Patients with Chronic Rhinosinusitis: A Population-Based Longitudinal Follow-Up Study

**DOI:** 10.3390/life13122238

**Published:** 2023-11-21

**Authors:** Dong-Kyu Kim, Hyunjae Yu

**Affiliations:** 1 Department of Otorhinolaryngology-Head and Neck Surgery, Chuncheon Sacred Heart Hospital, Hallym University College of Medicine, Chuncheon 24252, Republic of Korea; 2 Institute of New Frontier Research, Division of Big Data and Artificial Intelligence, Hallym University College of Medicine, Chuncheon 24252, Republic of Korea

**Keywords:** rhinosinusitis, sinusitis, glaucoma, cohort studies

## Abstract

Several studies have investigated the association between chronic rhinosinusitis (CRS) and ophthalmological complications. However, it remains uncertain whether CRS is independently associated with the development of normal tension glaucoma (NTG). Therefore, this retrospective cohort study aimed to investigate the prospective association between CRS and the increased incidence and risk of NTG using a representative population-based dataset. The selection of both the CRS and comparison groups was meticulously conducted through the propensity scoring method. The incidence and risk ratios of NTG were measured using person-years at risk and a weighted Cox proportional hazards model. We enrolled 30,284 individuals without CRS (comparison group) and 15,142 individuals with CRS. The NTG incidence rates were 1.19 and 0.81 in the CRS and comparison groups, respectively. The CRS group showed a significantly increased risk of subsequent development for NTG (adjusted hazard ratio = 1.41, 95% confidence interval = 1.16–1.72), regardless of the CRS subtype. Additionally, the risk of developing NTG was relatively higher in the first 2 years after CRS diagnosis. Moreover, a subgroup analysis revealed a higher risk of NTG in elderly female individuals with CRS. The present findings underscore the importance of monitoring and managing NTG risk in individuals with CRS, especially in elderly female patients.

## 1. Introduction

Chronic rhinosinusitis (CRS), one of the most common upper respiratory tract diseases, is characterized by persistent inflammation of the paranasal sinuses and nasal passage linings lasting 12 weeks or longer [1]. Clinically, the common sinonasal symptoms of CRS include congestion, nasal drainage, facial pressure/pain, and loss of smell. Thus, CRS adversely affects patients’ quality of life and reduces workplace productivity. According to epidemiological studies, the prevalence of chronic sinusitis is 5% in Canada, 12% in the United States, 11% in Europe, and 7% in South Korea [2,3,4,5]. Although it is a heterogeneous group of related disorders that share certain clinical and pathologic features, it is differentiated into subtypes, CRS with nasal polyps (CRSwNP) and CRS without nasal polyps (CRSsNP), depending on the presence or absence of nasal polyps during the rhinoscopy. Thus, these groups also show several different immunological features [6,7,8,9,10]. To date, various potential etiological and disease-modifying factors involved in CRS pathogenesis have been proposed, suggesting that chronic inflammation in CRS has a multifactorial origin [11,12].

Topical corticosteroids, with or without antibiotics, are the first-line therapies in patients with CRS [13]. A short course of oral corticosteroids alone or as a standard maintenance therapy is sometimes used to improve sinus symptoms or decrease NP size in patients with CRS. Surgery is typically reserved for cases where medical management fails. Notably, if patients with CRS avoid treatment for long periods, orbital and intracranial complications may occur [14]. These complications can require emergency surgery, hinder brain and eye function, and, in some cases, pose life-threatening risks [15]. Additionally, previous studies have shown that the respiratory distress condition is very closely correlated with increased intraocular pressure (IOP) [16,17]. Moreover, some cohort studies from Taiwan revealed a positive correlation between patients with CRS and the subsequent development of open-angle glaucoma [18,19].

Glaucoma is a progressive eye disease characterized by optic nerve damage that gradually worsens over time. In general, glaucoma can occur at any age, but it is known to be more common in older individuals and is one of the main causes of blindness in people aged over 60 years. Among the risk factors for glaucoma, IOP is the only modifiable factor. It is widely recognized that even in people with clinically normal IOP, the effective control of IOP significantly reduces the risk of developing glaucoma [20]. Glaucoma can be classified into four subtypes: open-angle, angle-closure, secondary, and normal tension glaucoma (NTG). The incidence and risk rates for each subtype of glaucoma also vary by race and country [21,22,23,24]. Previous epidemiological studies have indicated that NTG accounts for most open-angle glaucoma cases in East Asia [21]. Unlike what is observed in the Caucasian population, the rate of NTG in the East Asian population is very high, a phenomenon unique to East Asia [22,23,24].

NTG is characterized by progressive optic neuropathy with an IOP within the normal range. Its structural features and functional impairments are similar to those of primary open-angle glaucoma. However, unlike high-pressure glaucoma, NTG is believed to occur due to a complex interaction of multiple ocular and systemic factors, such as unstable vascular hemodynamics. This means that the risk factors for the development and progression of NTG include ocular factors, such as IOP, and systemic factors, such as vascular hemodynamics [25,26]. Altered vascular hemodynamics is also commonly observed in non-treated patients with CRS because chronic inflammation and angiogenesis are closely intertwined [27]. However, to date, few studies have investigated the possible link between CRS and an increased risk of NTG.

Therefore, we aimed to investigate the potential association between CRS and NTG in this study. Thus, we evaluated and compared the incidence rate and the risk ratio of NTG in patients with CRS compared to non-CRS patients using a representative population-based cohort dataset. It provides the entire medical history of a representative cohort sample (approximately one million South Koreans). We determined the incidence and risk for the primary endpoint between the target and comparative cohort groups using matched-cohort samples.

## 2. Materials and Methods

### 2.1. Cohort Dataset

In this study, we used a representative sample cohort dataset created using information from the National Health Insurance Service database in South Korea. These data have been organized into a cohort structure, facilitating longitudinal studies on rare diseases. South Korea has a health care system that benefits almost all of its citizens, which covers approximately 98% of the population. As a result, claims data include 46 million patients per year, equivalent to 90% of the entire population of South Korea. Therefore, the cohort dataset used in this study included patient diagnoses, treatment records, procedures, surgical histories, and prescription drug claims from virtually all healthcare providers across Korea, allowing us to conduct longitudinal, national, and population-based data analyses. The cohort dataset consisted of a representative sample reflecting approximately 2.2% of the Korean population, comprising a total of 1,025,340 adults. Additionally, this cohort dataset has proven to have an excellent reliability rating through a previously conducted reliability verification study [28,29]. Before study initiation, we obtained a waiver for the requirement for written informed consent because this cohort database did not include any certain clue or specific personal information regarding individual issues. Instead, data were provided to the investigator in a de-identified secondary data form. Therefore, the datasets generated and/or analyzed in the present study are not publicly available because of the policies of the Korean National Health Insurance Service. However, the authors confirm that the data supporting the findings of this study are available in the article.

### 2.2. Study Design/Participant Enrollment

**Figure 1:**
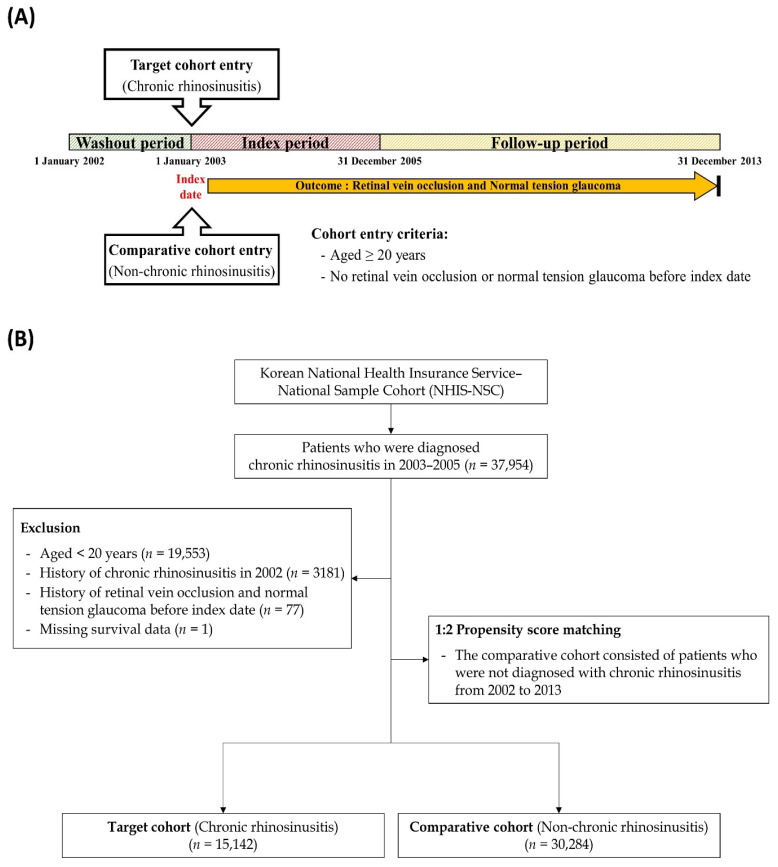
(**A**) Schematic illustration of the study design consisting of the washout period, index period, and follow-up period. (**B**) A brief illustration of the enrollment process of the participant cohort database in the present study.

This study was approved by the Institutional Review Board of Hallym College of Medicine, Chuncheon Sacred Hospital (protocol code 2019-10-009), and all authors claim that the study was conducted in accordance with the guidelines of the Declaration of Helsinki. To elucidate a potential link between CRS and NTG, we designed a retrospective cohort study based on a nationwide, representative cohort dataset. The study design is briefly described in Figure 1A. The first year of this cohort dataset was set as a washout period to exclude potential preexisting cases of NTG before the diagnosis of CRS. The operational definitions of the study end points were primary outcome events and all-cause mortality during the follow-up period. If the enrolled patients showed no primary outcome events or were still alive until the final day of the cohort dataset, they were censored at that time point. A schematic flow of participant enrollment in this study is presented in Figure 1B. To construct the target cohort, we identified patients with CRS using specific diagnostic codes based on the Korean Classification of Diseases, Fifth Edition, a modification of the International Classification of Disease and Related Health Problems, 10th revision (ICD-10). The CRS group included all patients who received hospitalization or outpatient treatment for an initial diagnosis of CRS (J32 [CRSsNP] and J33 [CRSwNP]) during the index period. It excluded patients who used biologic agents during the subsequent observation period as biologics can alter the disease course of CRS, especially CRSwNP. To create the comparative cohort, we selected non-CRS individuals from the remaining cohort registered in the database and then randomly matched non-CRS individuals to CRS patients using the propensity scoring methodology (two participants without CRS for each patient with CRS). We set gender, age, residence, income level, and comorbidities as independent variables and matched them between the two groups. In particular, we used the Charlson Comorbidity Index, the most widely used method in claims-based research, to match comorbidities. As this study is a big data study, we operationally defined CRS to improve the accuracy of CRS diagnosis as follows. First, if rhinosinusitis has been diagnosed more than twice within 3 months. Second, if it has become chronic, as confirmed through head and neck computed tomography. We excluded patients aged <20 years, those who died between 2003 and 2005, and those diagnosed with glaucoma before a CRS diagnosis. Additionally, patients who received systemic corticosteroids commonly administered for CRS, such as oral prednisolone and dexamethasone, for more than 4 weeks were also excluded from this study to eliminate the possibility of the development of steroid-induced glaucoma. To increase the analytical power of the study, we included patients diagnosed with CRS by rhinologists and those diagnosed with NTG by ophthalmologists. Eventually, 15,142 CRS patients and 30,284 non-CRS participants were enrolled in the present study.

### 2.3. How to Process Statistics for This Study

To calculate the overall incidence of NTG, we divided the number of patients diagnosed with NTG by 1000 person-years. We measured the period between the date of patient enrollment and each patient’s individual endpoint. Next, we used the Cox proportional hazards regression to determine whether CRS could increase the hazard ratio of incident-specific disease events. These statistics were expressed as hazard ratios (HRs) and 95% confidence intervals (CIs). We also reported both crude and weighted HRs with 95% CIs. Finally, the Kaplan–Meier method was used to describe the cumulative probability of developing NTG over the entire follow-up period in the study and control groups. R software (version 4.0.0) was used for all statistical analyses described here (R Foundation for Statistical Computing, Vienna, Austria), and statistical significance was determined at a *p* value < 0.05.

## 3. Results

### 3.1. Group Matching between the Target and Comparative Cohorts

During the entire follow-up period, we assessed the overall incidence of NTG events in CRS patients and control participants (comparison group, non-CRS). The characteristics of the target and comparative cohorts are summarized in Table 1. Each independent variable, including sex, age, residential area, household income, or comorbidities, was similar between the target and control cohorts. Additionally, to assess the effectiveness of propensity score matching for these five covariates, we employed a balance plot methodology before and after group matching (Table 2). Our analysis indicated considerably enhanced profiles after group matching. Based on these findings, we concluded that the two cohort datasets were appropriately matched for each covariate.

### 3.2. Overall Incidence Rate of Normal Tension Glaucoma

We found a total of 139,005.3 person-years and an incidence rate of 1.19 for NTG in patients with CRS, whereas there were 286,913.3 person-years and an incidence rate of 0.81 for NTG in comparison participants. Additionally, in the analysis of the CRS subtypes, the incidence rates of the subsequent development of NTG events per 1000 person-years were 1.15 in the CRSsNP group (*n* = 14,124) and 1.81 in the CRSwNP group (*n* = 1018) compared to the control group (Table 3).

### 3.3. Risk Rate of Normal Tension Glaucoma

**Figure 2:**
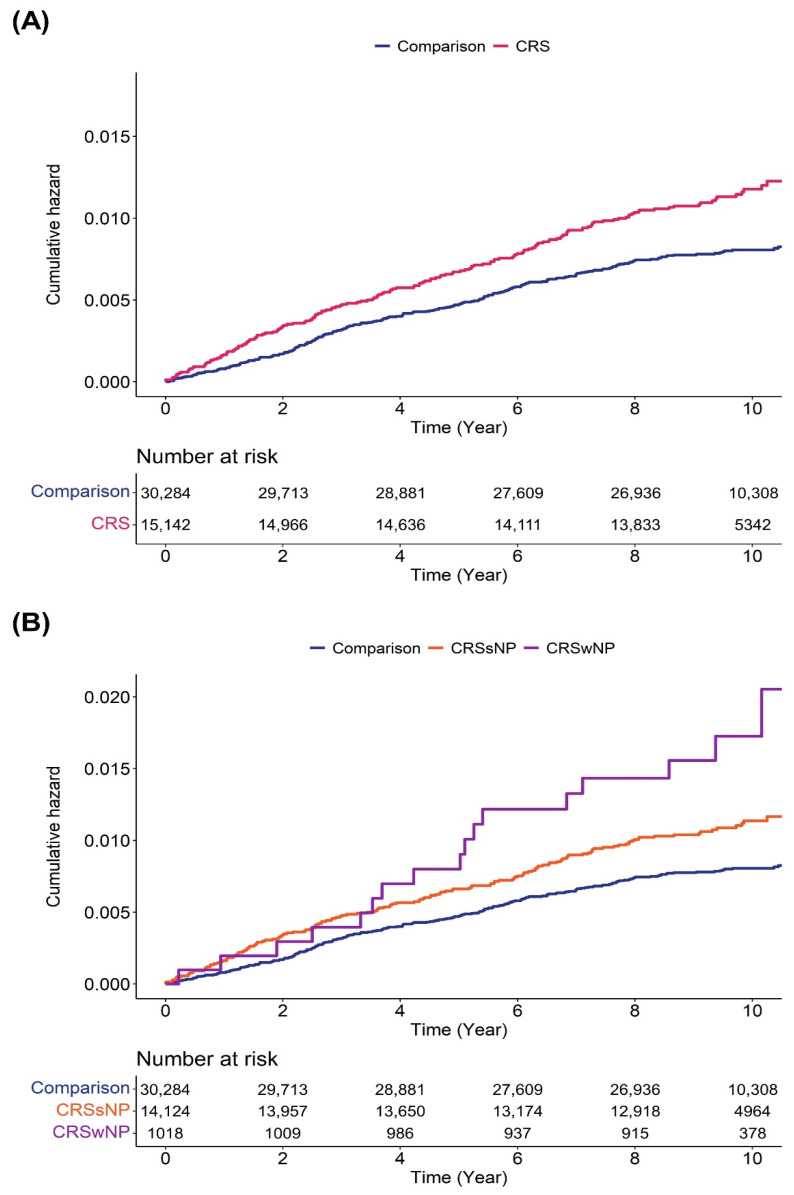
Cumulative hazard plot of incidental normal tension glaucoma events according to the subtype of chronic rhinosinusitis. (**A**) CRS, chronic rhinosinusitis; (**B**) CRSsNP, chronic rhinosinusitis without nasal polyp and CRSwNP, chronic rhinosinusitis with nasal polyp.

To evaluate the risk ratio of NTG events in patients with CRS, we measured crude and weighted HRs with a 95% CI using univariate and multivariate Cox regression models. After adjusting for all independent variables, we observed that the risk rate of incident NTG events was 1.41 times higher (95% CI, 1.16–1.72) in patients with CRS compared to the comparison group (Table 4). Furthermore, we analyzed the adjusted HR for incident NTG events according to the CRS subtype. After adjusting for all independent variables, we found that CRSsNP patients displayed a significantly increased risk ratio for the subsequent development of NTG events (adjusted HR = 1.37, 95% CI: 1.12–1.69), while CRSwNP patients exhibited an adjusted HR of 1.88 (95% CI: 1.14–3.08) for incident NTG events during the follow-up period. Additionally, we evaluated the adjusted HR of incident NTG events over time (Table 5). This analysis revealed that the risk ratio for the subsequent development of NTG events was relatively high in the first 2 years after a CRS diagnosis. Although the risk decreased after this initial period, the overall risk ratio remained significantly higher throughout the follow-up period. Figure 2 illustrates the Kaplan–Meier survival curves with log-rank tests for the cumulative hazard plot of NTG events between the comparison and CRS groups. The results of the log-rank test indicated that patients diagnosed with CRS experienced NTG events more frequently than those without CRS during the 10-year follow-up period, regardless of the *specific* CRS subtypes, such as CRSsNP and CRSwNP.

### 3.4. Subgroup Analysis

Next, we performed subgroup analyses to examine the association between CRS and an increased risk of NTG according to sex and age. After adjusting for other variables, we observed that female CRS patients displayed a higher likelihood of developing NTG (adjusted HR = 1.58, 95% CI = 1.21–2.05), whereas male CRS patients did not exhibit a significant risk of developing NTG events (adjusted HR = 1.22, 95% CI = 0.90–1.66) (Table 6). Moreover, we observed that the adjusted HR for developing NTG events in patients with CRS was significantly higher in the age groups under 45 years and over 64 years, but there was no significantly increased risk in the 45–64 age category (<45 years: adjusted HR = 1.60, 95% CI = 1.13–2.25; 45–64 years: adjusted HR = 1.17, 95% CI = 0.87–1.58; >65 years: adjusted HR = 1.74, 95% CI = 1.13–2.69) (Table 7). However, when we further examined the adjusted HR according to the age category in female patients with CRS, we found that only the elderly group (>64 years) had a significantly increased risk of subsequent development of NTG events, with a 2.29-fold higher risk (95% CI = 1.33–3.97) (Table 8). This finding indicates that the association between NTG events and older age in female patients with CRS may not be coincidental.

## 4. Discussion

CRS is a commonly diagnosed upper respiratory inflammatory disease that causes a reduced quality of life, as CRS also causes various related complications. Therefore, we examined the association with NTG in patients with CRS among various comorbidities. To the best of our knowledge, this is the first study to examine whether CRS could increase the risk of NTG using a population-based cohort dataset. This study revealed that CRS was associated with an increased risk of developing NTG. Notably, we found that the risk of NTG was relatively high within the first 2 years after a CRS diagnosis. Although the risk of NTG decreased after this initial period, the significant association between CRS and an increased NTG risk persisted over time. These results imply that the association between CRS and subsequent development of NTG is unlikely to be coincidental. Additionally, in the subgroup analysis, we found that elderly female patients with CRS had a higher risk of developing NTG than elderly male patients. Based on this analysis, we can suggest the possibility that elderly female patients may be relatively vulnerable to developing NTG complications. However, as this was a clinical observational study, further laboratory investigations are required to prove the precise underlying pathophysiological mechanisms connecting these two diseases.

Several studies have reported a positive correlation with an increased risk of glaucoma in patients with CRS. For example, one study showed an increased risk of developing open-angle glaucoma during the follow-up period in CRS patients compared to non-CRS patients [18]. Another study highlighted that surgery-associated CRS contributed to a significant risk ratio in both the open-angle glaucoma (adjusted HR = 2.244) and NTG (adjusted HR = 2.127) subgroups, but not in the angle-closure glaucoma subgroup. Consistently, the present investigation also demonstrated an increased risk ratio for incident NTG events in all CRS patients, with an adjusted HR of 1.41 over a 10-year follow-up period [19]. Moreover, several studies hypothesized that ostial obstruction in CRS could limit airflow, leading to hypoxia, which could contribute to retinal ischemia, a factor associated with glaucoma development [30,31,32]. Currently, CRS is divided into two phenotypes. Numerous studies have suggested that CRSsNP has a primary type 1 immune response accompanied by a mixture of immune cells, whereas type 2 inflammation accompanied by increased eosinophil infiltration is predominantly found in CRSwNP [10,33,34,35]. Notably, CRSwNP has a more severe inflammatory state in the paranasal mucosa, contributing more strongly to the development or exacerbation of hypoxic conditions [7,36]. In line with these findings, our study revealed that the risk ratio for subsequent NTG development was substantially higher in CRSwNP (adjusted HR = 1.88, 95% CI: 1.14–3.08) than in CRSsNP (adjusted HR = 1.37, 95% CI: 1.12–1.69).

However, unlike regular glaucoma, NTG is a subtype of open-angle glaucoma that presents with an IOP of less than 21 mmHg. Its diagnosis is based on ophthalmological signs and visual field defects in the absence of disc abnormalities or other causes of visual field loss. Consequently, there remains an ongoing debate regarding whether NTG should be categorized within the scope of primary open-angle glaucoma or recognized as a unique disease entity. Nevertheless, NTG exhibits several differences from primary open-angle glaucoma, such as risk factors for NTG development independent of IOP and a unique disease course [37]. Although IOP is considered the most significant risk factor for NTG, several other factors have been shown to influence its development and progression, including peripheral vascular disease, hemodynamic crisis, low blood pressure, and carotid insufficiency [38,39,40,41]. Previous studies have also suggested that conditions triggering vasospasm, such as migraines and Raynaud’s phenomenon, are risk factors for developing NTG [42,43]. Additionally, one study demonstrated that migraines, a possible surrogate marker for vasospasm, and female sex may contribute to the risk of NTG progression [44]. Specifically, that study revealed that female individuals with migraines were at an increased risk of NTG progression [44]. Sinus headaches are usually associated with migraines or other forms of headache because they induce pain and pressure in the face and sinuses and can cause nasal symptoms [45,46]. Migraines can be accompanied by various nasal signs and symptoms because they involve the autonomic nervous system. Moreover, in studies examining sex ratios, it was noted that women were approximately three times more likely than men to have migraines, and female sex was a risk factor for NTG [47,48]. Consistent with these findings, we found that elderly female patients with CRS had a higher risk of developing NTG.

Our study has the following limitations. First, the cohort database lacks details related to CRS severity, such as the Lund–Macay score and the Sinus Outcome Test 22 questionnaire. Additionally, the operational definition of CRS relied primarily on ICD-10 diagnosis codes, which are less accurate than diagnoses based on medical chart data, including the patient’s medical history and nasal examination results. Therefore, a certain level of misclassification bias is likely to have occurred in this study. Furthermore, our investigation might also have a surveillance bias, as CRS patients with reduced quality of life are likely to visit healthcare facilities frequently, potentially resulting in the earlier detection of NTG unrelated to their CRS status owing to increased exposure to health services. This could introduce bias into the incidence and risk measures, as it may enable the early detection of possibly unrelated conditions. To minimize this problem, all patients with CRS and NTG should be diagnosed by board-certified rhinologists and ophthalmologists, respectively. Second, NTG presents diagnostic and therapeutic challenges for ophthalmologists, which could lead to an underestimation of the number of NTG diagnoses included in the cohort dataset. Additionally, information on ophthalmologic examination results that could help assess the severity of glaucoma or the accuracy of the diagnosis code was not provided. Third, depending on the design of this study, it is unknown whether our findings indicate a causal relationship between the two diseases or a simple temporal association between them. However, despite these limitations, several previous studies examining the relative association between the two diseases have used the same cohort dataset as ours [49,50,51,52]. Further clinical and experimental studies are required to confirm the possible link between these two diseases. Finally, family history, genetic predisposition, radiographic findings regarding severity, and the use of intranasal steroid therapy can all affect the potential development of glaucoma, which might introduce confounding bias into our findings. To mitigate these limitations, we enrolled only patients diagnosed by rhinologists and ophthalmologists and included a 1-year washout period in our study design. Additionally, numerous prior investigations failed to establish a clear relationship between intranasal steroid therapy and IOP [53,54,55]. Moreover, we matched the CRS and comparative cohort groups using propensity scores based on several important independent covariates and conducted a relatively long follow-up period to test the robustness of our findings. Furthermore, our analysis of HRs over time consistently indicated significantly increased HRs for NTG events in patients with CRS throughout the 10-year follow-up period.

## 5. Conclusions

This study investigated whether CRS affects the subsequent development of NTG using a population-based longitudinal approach. We found that CRS may be involved in the subsequent development of NTG, regardless of the CRS subtype, and elderly female patients with CRS may be more likely to develop NTG. Therefore, our findings suggest that healthcare providers take specific precautions to detect NTG early in patients with CRS.

## Figures and Tables

**Table 1:** Detailed characteristics of the enrolled study participants.

Variables	Comparison (*n* = 30,284)	CRS (*n* = 15,142)	*p*-Values
**Sex**			1.000
Male	12,172 (40.2%)	6086 (40.2%)	
Female	18,112 (59.8%)	9056 (59.8%)	
**Ages (years)**			1.000
<45	18,500 (61.1%)	9250 (61.1%)	
45–64	9298 (30.7%)	4649 (30.7%)	
>64	2486 (8.2%)	1243 (8.2%)	
**Residence**			1.000
Seoul	8100 (26.7%)	4050 (26.7%)	
Second area	7990 (26.4%)	3995 (26.4%)	
Third area	14,194 (46.9%)	7097 (46.9%)	
**Household income**			1.000
Low (0–30%)	5610 (18.5%)	2805 (18.5%)	
Middle (30–70%)	11,270 (37.2%)	5635 (37.2%)	
High (70–100%)	13,404 (44.3%)	6702 (44.3%)	
**Comorbidities status (CCI)**			1.000
0	18,938 (62.5%)	9469 (62.5%)	
1	6860 (22.7%)	3430 (22.7%)	
≥2	4486 (14.8%)	2243 (14.8%)	

Comparison among participants without CRS; Seoul, the largest metropolitan area; second area, other metropolitan cities; third area, other areas; CCI, Charlson Comorbidity Index.

**Table 2:** Baseline data before and after propensity score-matching.

Variables	Before Propensity Score Matching			After Propensity Score Matching		
	Comparison (*n* = 653,882)	CRS (*n* = 15,142)	*p* Value	Comparison (*n* = 30,284)	CRS (*n* = 15,142)	*p* Value
**Sex**			<0.001			1.000
Male	332,560 (50.9%)	6086 (40.2%)		12,172 (40.2%)	6086 (40.2%)	
Female	321,322 (49.1%)	9056 (59.8%)		18,112 (59.8%)	9056 (59.8%)	
**Ages (years)**			<0.001			1.000
<45	389,393 (59.6%)	9250 (61.1%)		18,500 (61.1%)	9250 (61.1%)	
45–64	185,964 (28.4%)	4649 (30.7%)		9298 (30.7%)	4649 (30.7%)	
>64	78,525 (12.0%)	1243 (8.2%)		2486 (8.2%)	1243 (8.2%)	
**Residence**			<0.001			1.000
Seoul	137,361 (21.0%)	4050 (26.7%)		8100 (26.7%)	4050 (26.7%)	
Second area	169,618 (25.9%)	3995 (26.4%)		7990 (26.4%)	3995 (26.4%)	
Third area	346,903 (53.1%)	7097 (46.9%)		14,194 (46.9%)	7097 (46.9%)	
**Household income**			<0.001			1.000
Low (0–30%)	164,474 (25.2%)	2805 (18.5%)		5610 (18.5%)	2805 (18.5%)	
Middle (30–70%)	250,525 (38.3%)	5635 (37.2%)		11,270 (37.2%)	5635 (37.2%)	
High (70–100%)	238,883 (36.5%)	6702 (44.3%)		13,404 (44.3%)	6702 (44.3%)	
**CCI**			<0.001			1.000
0	501,704 (76.7%)	9469 (62.5%)		18,938 (62.5%)	9469 (62.5%)	
1	83,731 (12.8%)	3430 (22.7%)		6860 (22.7%)	3430 (22.7%)	
≥2	68,447 (10.5%)	2243 (14.8%)		4486 (14.8%)	2243 (14.8%)	

Comparison, subjects without CRS; Seoul, the largest metropolitan area; second area, other metropolitan cities; third area, other areas; CCI, Charlson comorbidity index.

**Table 3:** Incidence rate per 1000 person-years of normal tension glaucoma according to the CRS subtype.

Variables	N	Case	Person Year	Incidence Rate
Comparison	30,284	233	286,913.3	0.81
CRS	15,142	166	139,005.3	1.19
CRSsNP	14,124	149	129,638.8	1.15
CRSwNP	1018	17	9366.5	1.81

CRS, chronic rhinosinusitis; CRSsNP, chronic rhinosinusitis without nasal polyps; CRSwNP, chronic rhinosinusitis with nasal polyps.

**Table 4:** Risk ratios of normal tension glaucoma according to the CRS subtype.

Variables	Unadjusted HR (95% CI)	Adjusted HR (95% CI)	*p*-Values
Comparison	1.00 (ref)	1.00 (ref)	
CRS	1.44 (1.18–1.75) ***	1.41 (1.16–1.72) ***	<0.001
CRSsNP	1.38 (1.13–1.70) **	1.37 (1.12–1.69) **	0.002
CRSwNP	2.19 (1.34–3.58) **	1.88 (1.14–3.08) *	0.013

CRS, chronic rhinosinusitis; CRSsNP, chronic rhinosinusitis without nasal polyps; CRSwNP, chronic rhinosinusitis with nasal polyps; HR, hazard ratio; CI, confidence interval. * *p* < 0.05, ** *p* < 0.010, and *** *p* < 0.001.

**Table 5:** Changes in the risk of normal tension glaucoma according to the observation period.

Time (Year)	Normal Tension Glaucoma	
	Number of Events	Adjusted Hazard Ratio (95% CI)
1	25	2.08 (1.19–3.64) *
2	51	1.98 (1.35–2.93) ***
3	70	1.46 (1.07–1.99) *
4	86	1.43 (1.08–1.88) *
5	100	1.41 (1.09–1.82) **
6	116	1.34 (1.06–1.70) *
7	136	1.39 (1.12–1.74) **
8	151	1.38 (1.12–1.70) **
9	156	1.37 (1.11–1.68) **
10	164	1.41 (1.15–1.72) ***
11	166	1.41 (1.16–1.72) ***

CI, confidence interval. * *p* < 0.05, ** *p* < 0.010, and *** *p* < 0.001.

**Table 6:** Risk ratios of normal tension glaucoma by sex between non-CRS and CRS.

Sex	Males		Females	
	Comparison	CRS	Comparison	CRS
**Normal tension glaucoma**				
Crude HR (95% CI)	1.00 (ref)	1.24 (0.91–1.68)	1.00 (ref)	1.61 (1.24–2.09) ***
Weighted HR (95% CI)	1.00 (ref)	1.22 (0.90–1.66)	1.00 (ref)	1.58 (1.21–2.05) ***

CRS, chronic rhinosinusitis; HR, hazard ratio; CI, confidence interval. *** *p* < 0.001.

**Table 7:** Risk ratios of normal tension glaucoma by ages between non-CRS and CRS.

Ages	<45 Years		45–64 Years		>64 Years	
	Comparison	CRS	Comparison	CRS	Comparison	CRS
**Normal tension glaucoma**						
Crude HR (95% CI)	1.00 (ref)	1.59 (1.13–2.25) **	1.00 (ref)	1.18 (0.87–1.59)	1.00 (ref)	1.74 (1.13–2.69) *
Weighted HR (95% CI)	1.00 (ref)	1.60 (1.13–2.25) **	1.00 (ref)	1.17 (0.87–1.58)	1.00 (ref)	1.74 (1.13–2.69) *

CRS, chronic rhinosinusitis; HR, hazard ratio; CI, confidence interval. * *p* < 0.05 and ** *p* < 0.010.

**Table 8:** Risk ratios of normal tension glaucoma in female patients with CRS by age.

Normal Tension Glaucoma	<45		45–64		>64	
	Comparison	Female CRS	Comparison	Female CRS	Comparison	Female CRS
Crude HR (95% CI)	1.00 (ref)	1.61 (1.00–2.60)	1.00 (ref)	1.29 (0.87–1.91)	1.00 (ref)	2.31 (1.34–3.99) **
Weighted HR (95% CI)	1.00 (ref)	1.61 (1.00–2.59)	1.00 (ref)	1.29 (0.87–1.91)	1.00 (ref)	2.29 (1.33–3.97) **

CRS, chronic rhinosinusitis; HR, hazard ratio; CI, confidence interval. ** *p* < 0.010.

## Data Availability

The authors confirm that the data supporting the findings of this study are available within the article.

## Abbreviations

CRS	Chronic rhinosinusitis
CRSwNP	Chronic rhinosinusitis with nasal polyp
CRSsNP	Chronic rhinosinusitis without nasal polyp
CI	Confidence interval
HR	Hazard ratio
ICD-10	International Classification of Disease and Related Health Problems, 10th revision
NTG	Normal tension glaucoma
